# Supplementary figures and images for: Using machine learning-based variable selection to identify hydrate related components from FT-ICR MS spectra
Source: PLoS One. 2022 Aug 17;17(8):e0273084. doi: 10.1371/journal.pone.0273084 (PMC9385063; doi:10.1371/journal.pone.0273084)

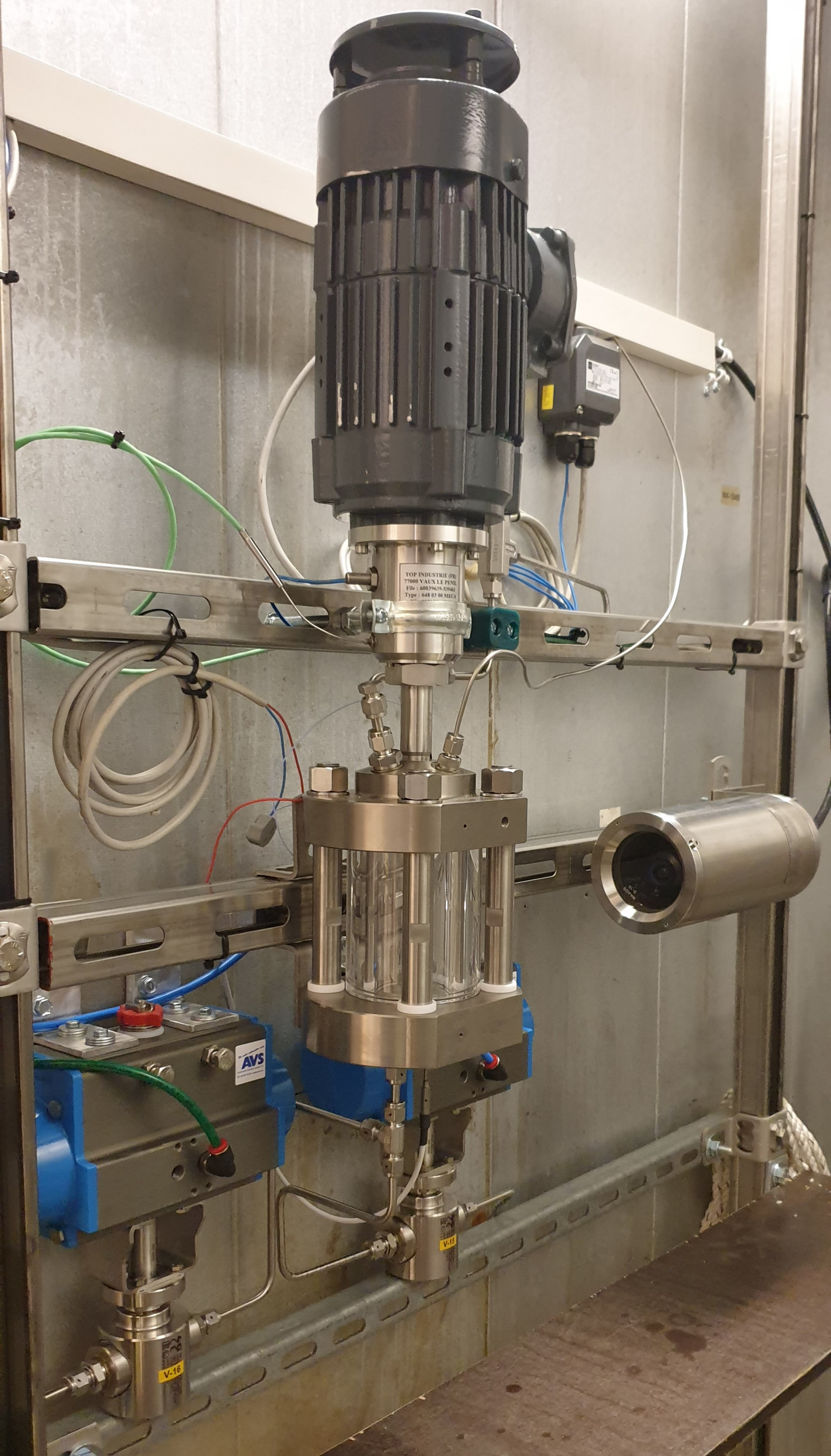

Supplement: S1 Fig — Picture of the autoclave used for the hydrate formation and spiking experiments. It consists of a sapphire cell between two titanium grad II flanges. Pressure, temperature and conductance is measured inside the sapphire cell. A motor is mounted above the cell driving a stirrer through a magnetic connection. (PDF) [file pone.0273084.s001.pdf]
