# Supplementary material for: Using machine learning-based variable selection to identify hydrate related components from FT-ICR MS spectra
Source: PLoS One. 2022 Aug 17;17(8):e0273084. doi: 10.1371/journal.pone.0273084 (PMC9385063; doi:10.1371/journal.pone.0273084)
Supplement: Attachment — Submitted filename: Referee comments.docx [file pone.0273084.s004.docx]

PONE-D-22-13309

Using machine learning-based variable selection to identify

hydrate related components from FT-ICR MS spectra

**SUMMARY**

The authors address an important problems in oil and gas production, the formation of solid gas hydrates. They do it with an interesting experimental design (repeated enrichment of possible hydrat constitutents in three different oil raw materials,) in an advanced but already tested experimental set-up (high-pressure reactor and - filter, with temperature control).

The resulting samples are analyzed using an advanced multi-channel chemical instrument (FT-ICR MS), resulting in Big Data measurements.

In order to find the variation patterns in the data that seem to be related to hydrate formation, they	enploy a range of data analytic methods coming from different scientific cultures: Generic PCA and raw-data difference plots, as well as a range of multivariate classification methods	to distinguish between different enrichment sample groups. Decision Trees (DTs), Random forest (RF) , Ensemble learning and LASSO from the statistical learning and AI cultures and Partial Least Squares Discriminant Analysis (PLS-DA) from Chemometrics).

In each case, the focus has been to identify the properties of oil constituents that are important for hydrate formation in the three oil raw materials. They do so by variable selection, to identify molecular species that seem to be enriched during the successive hydrate- accumulation experiments.

Preliminary PCA showed that the successive hydrate- accumulation gradually changed the hydrate composition, as expected. Simple plots of difference spectra pointed towards some suspects in the formation of gas hydrates.

The different multivariate classification methods were then compared with respect to their abililty to give consistent selection of hydrate-relevant MS peaks in subsets of the available samples. The methods differed considerably in this respect. Many of the statistical/AI based methods were unable to find consistent variable selections, at least as implemented for this paper (presumably a standard implementation). In contrast, the PLS-DA and Random Foerst methods were found to give clearly consistent variable selection. Between them the PLS-DA showed higher classification accuracy wrt enrichment sample group.

The variables where the PLS-DA consistently indicated an ability to give gas hydrates, was studied wrt their most probable molecular characteristics: From the 25 PLS-DA reduced sample sets, 44 variables appeared to be important in every model. Some of their molecular properties were determined, in an attempt to identify molecules related to hydrate formation. Some of the variables were identified as possible asphaltenic structures which could be related to the positive WI for the oils.

**Referee’s assessment of the paper:**

The paper addresses a real problem, with advanced tools and a cross-cultural willingness to compare data analysis methods. I believe their conclusions are warranted, given their results.

The title is relevant. The abstract is informative and compact. The Introduction needs some clarification. Wrt to Materials and Methods, I cannot judge the oil-relevant or MS-relevant descriptions. But the data analytic descriptions need some clarification.

The Results and the Discussion sections the also need a few clarifications.

The Conclusions make sense, based on the Results, and correspond well to the paper’s purpose and title. The references seem relevant.

I like this paper.

**MAJOR ISSUES**

None.

**MINOR ISSUES**

**Introduction**

Please comment: The water phase was made by tap water with 3.5% NaCl. It that reprsentative for the water composition encoundered in gas-and oil-production? Ocean water has Na^+^ and Mg^++^ cations in about equal concentrations <https://centerforsurfresearch.org/chemical-composition-of-seawater/> ). Could it be that the cross-linking ability of divalent cations like Mg^++^ in charged structures is relevant for the formation of solid hydrates?

**Methods**

It is highly commendable that the authors compare classification methods from different data analytical cultures. But make sure that all the methods are described with the same mathematical symbols for the same phenomena, as far as possible.

Please mention that while variable selection can be important in order to get rid of irrelevant variables, the process of variables selection can consume a lot of degrees of freedom (DF) and thus lead to overfit. Please describe how each of the classification methods are implemented in order to reduce overfit (cross-validation/test-set/DF-correction etc ?).

The bilinear model descriptions(PCA, PLSDA) lack information about how the variables have been centered and scaled. May I presume that each variable has been standardized to mean=0 and std.dev.=1? If so, write that in the text!

I am not impressed with the bilinear method descriptions (PCA, PLS-DA, VIP). They are generanlly a bit too brief to be understood and reproduced by people outside the chemometric culture. More-over the PLS-DA description is incomplete and must be corrected. As it stands, X and Y seem to be uncentered and scaled. Worse, X is modelled by T and Y by U, but U and T are not related.

I propose these alternatives:

Please explain more clearly by “determine the sample class “ in “When Y is categorical and the problem is classification, linear discriminant analysis is used to determine the sample class as shown by Barker et al. [35].”

VIP

The index notation used for VIP is sloppy. Index upper limit **a** is a bit inconsistent with the PLSR symbol for rank, **A**. Index **K** and **k** must be defined, and the paranthesis must be closed:

Moreover, please give a brief verbal description of the *logic* behind VIP.

Please describe the **statistical threshold -values** used for selecting peaks in the different classification methods. Are you comparing the different classification methods in a fair way, or using widely different statistical test levels?

**Results**

Relative scaling of the MS variables:

Important: Unless you standardized the MS variables in the PCA and PLS-DA models, please repeat all the analyses after having done so. Or, alternatively, divided each MS peak by the corresponding MS peak in the 0-sample, for each oil. Do you still get similar method-ranking and resulting m/z lists?

Suggestion: You might also want to try to use the fourth root of the MS-variables, in order to avoid that the classifications are dominated by a few extreme outlier samples.

How did you define the Accuracy?

Important: In Figure 7: Please define how the criterion **accuracy** was calculated. Is it related to the description “most *accurately* predicts the differences between the bulk samples and the hydrate samples, and therefore selects the variables with the highest probability of being related to hydrate formation.”? If so, then write that.

Preliminary analyses

Only 3 out of 5 peaks in Table 2 were found in Table 4. I think you should discuss that briefly, even though it is just preliminary.

**OTHER COMMENTS**

Layout problem:

Table 3 is printed *after* Table 4.
