# Supplementary material for: Using machine learning-based variable selection to identify hydrate related components from FT-ICR MS spectra
Source: PLoS One. 2022 Aug 17;17(8):e0273084. doi: 10.1371/journal.pone.0273084 (PMC9385063; doi:10.1371/journal.pone.0273084)
Supplement: Attachment — Submitted filename: Rebuttal_letter_EG.docx [file pone.0273084.s005.docx]

Dear Dr. Banoub,

Please find our answers to the points raised by the reviewers below.

**Reviewer 1**

The water phase of NaCl was chosen as it contains only mono-valent ions. This simplifies the water chemistry, avoiding possible unwanted reactions by bi-valent ions such as Ca^2+^. We have included this under the “Fluid systems” section of the experimental procedure.

For the linear methods, PCA, PLS-DA and LASSO, the data was standardised and mean centered to st.dev of 1 and mean of 0 before the classification and variable selection. This has now been specified in the text under the “Data analysis” section of the experimental procedure. All the methods have been validated using training and test set validation, with 25 different test set splits, as there was a difference in the accuracy between different compositions of training and test sets. Variable selection is prone to overfitting as it consumes degrees of freedom, but this is remedied by the test set, this has now been commented on in the discussion. The performances of the models are evaluated based on all the features not the reduced models. For all methods apart from PLS-DA, the variables are selected based on the feature importance attributes of the model. As PLS-DA does not have a built-in estimator for feature importance, VIP is used. The statistical threshold should thereby be comparable, as all models are optimised similarly.

The accuracy is defined as the fraction of correctly classified samples. This has been added to the text under the “Variable selection” section of the results.

The authors are grateful for the suggestion for the linear methods, PCA and PLS, and their descriptions have been updated accordingly. The section regarding PLS for classification has been amended. Additionally, the section on VIP has been rewritten entirely to better describe the method and the issues with the formula has been rectified.

**Reviewer 2**

During the FT-ICR MS analysis we are collecting 220 spectra for each sample. The spectra from the FT-ICR MS are then combined into one data matrix using Profile Analysis. A section “Data treatment” has been added to the experimental part describing how the data was treated in Profile Analysis, with all settings included. This data matrix is referred to as a bucket table, and it is the data that is being used for all of the data analysis in the paper. The data are solid, analysed in the same fashion as other crude oil studies using FT-ICR MS. The FT-ICR MS is run in mFT (magnitude Fourier Transform) mode.

Figures 4-6 are not mass spectra; they are showing the mass spectra for the sample with spiking level 0 subtracted from the remaining spiking levels for each of the oils. They are therefore the differences between two mass spectra. “Raw spectra” has been changed to “mass spectra” to further illustrate this. The Figures have been made larger and redundant white space in the figures has been removed.

For oxygen species that contain acidic or carboxylic groups it is true that they are better recorded in negative mode. However, this is not correct for all oxygen species, PEG, PPG or other compounds that have ether or amide bonds. Asphaltenes for example are well characterised by positive mode as well as, hydroxyl groups and amines. For example, [Qian et al](https://pubs.acs.org/doi/10.1021/ef000255y). showed that ESI(+) FT-ICR MS was able to identify more than 3000 chemical formulas of nitrogen containing aromatic compounds.

The HPLC is only used as a sample introduction device without a column. The 100 µL are injected over a period of 10 minutes with a flow of 10 µL per minute and 220 spectra are collected for each sample. This has been specified in the text under the “FT-ICR MS analysis” section of the experimental procedure.

Gas hydrates consist of the crude oils and their behaviour is dependent on the composition of the oils. In this paper the aim was to identify compounds related to hydrate formation by analysis of oil samples from hydrates. The interest is therefore of the differences in chemical composition between hydrates and bulk samples.

The wetting index is a measure of the emulsion inversion point with and without hydrates. This is specified under the section “Wetting index experiments” which includes references if the reader wishes a deeper understanding of the procedure. The authors are unclear on what is meant by “other engineering terms”, but have added the definition for water cut to the experimental section.

The variable with m/z 156.44 is an ion with charge 3, meaning that the molecular formula that was stated in the table is for the original molecule and not for this ion. Some of the variables are altered, see explanation of why in the next section. The remaining molecular formulas have been re-evaluated and some are changed to more plausible formulas. The molecular formulas for all variables have been added to the tables and the adduct of the molecule. The formulas are determined as the best fitting plausible mass using the DataAnalysis software. As mentioned in the discussion, the molecular formulas are only suggestions and the exact structures need to be determined experimentally.

**Additional alterations**

After submission of the paper, we discovered that there was an error in the code for the PLS-DA model. The PLS-DA has been recalculated with 20 components and Figure 7, Table 3 and Table 4 have been updated accordingly. For the new PLS-DA model, 132 variables were selected and 26 variables were selected in all of the 25 models. Two of the variables were stemming from the same peak and two were isotop peaks, resulting in a total of 23 variables. Table 4 shows the updated variables, 17 of the variables were the same as in the previous version of the paper. The VIP was calculated for all 25 test sets with 20 components in the PLS-DA model, and the results were plotted. This figure is added as Supporting information 2, and shows the curve flattening around 5, which was then selected as the threshold for VIP during the variable selection. This has now been specified in the text. The changes made, does not alter the conclusion of the work.

Some alterations have been made to the text, removing unnecessary information and repetitions to create a better flow. Updates to fit the style requirements have been done. This is shown in the document with track changes. The financial disclosure stated in the paper is the correct one, and it reads: “*The authors thank the Norwegian Research Council, Equinor ASA, OMV (Norge) AS, Wintershall DEA Norge AS and TotalEnergies for funding. This work is a part of the Knowledge-Building Project for Industry (PETROMAKS 2), Project number: 294636 “New Hydrate Management: New understanding of hydrate phenomena in oil systems to enable safe operation within the hydrate zone*”.

Thank you for considering our paper, please let us know if further revisions are required.

Sincerely,

Elise Lunde Gjelsvik
PhD Candidate, Faculty of Science and Technology,
Norwegian University of Life Sciences,
[elise.lunde.gjelsvik@nmbu.no](mailto:elise.lunde.gjelsvik@nmbu.no)
Telephone: +47 40469707
